# The relationship between exercise induced bronchial obstruction and health related quality of life in female and male adolescents from a general population

**DOI:** 10.1186/s12890-016-0226-0

**Published:** 2016-04-27

**Authors:** Henrik Johansson, Katarina Norlander, Christer Janson, Andrei Malinovschi, Leif Nordang, Margareta Emtner

**Affiliations:** Neuroscience/Physiotherapy, Uppsala University, Uppsala, Sweden; Surgical Sciences: Otolaryngology and Head & Neck Surgery, Uppsala University, Uppsala, Sweden; Medical Sciences, Lung- allergy- and sleep research, Uppsala University, Uppsala, Sweden; Medical Sciences, Clinical Physiology, Uppsala University, Uppsala, Sweden

**Keywords:** Adolescents, Exercise-induced bronchoconstriction, Health-related quality of life, Physical activity

## Abstract

**Background:**

Previous studies have observed that exercise-induced bronchoconstriction (EIB) is more common and more strongly related to exercise related breathing problems in female adolescents than male adolescents. However, few studies have investigated the association between EIB and health related quality of life (HRQoL) from a gender perspective. The aim of this study was to examine the association between EIB and HRQoL and physical activity level in female and male adolescents with and without EIB.

**Methods:**

From a population based study on exercise-induced breathing problems among adolescents (13–15 years, *n* = 3838) a cross sectional study with prospective data collection was carried out in a random subsample of 140 adolescents. The subjects in the sample were tested for EIB with a standardised exercise test, of which 49 adolescents were tested positive. HRQoL was assessed with the Pediatric Quality of Life Inventory (PedsQL) and the adolescents’ physical activity levels were measured objectively with accelerometer.

**Results:**

The female subjects with EIB reported a lower HRQoL, both in total score (*p* = 0.03) and physical functioning score (*p* = 0.009) and had a lower baseline FEV_1_ compared to females without EIB (88 vs. 94 % predicted, *p* = 0.001). No differences were found in HRQoL or baseline lung function between males with or without EIB. There were no differences in minutes of moderate to vigorous physical activity per day between females or males with and without EIB.

**Conclusion:**

In a general population, the female adolescents with EIB had lower HRQoL and poorer baseline lung function compared to counterparts without EIB. These differences were not observed in males. EIB does not appear to affect the level of daily physical activity neither in females nor males.

## Background

Exercise-induced bronchoconstriction (EIB), defined as the transient narrowing of the lower airways following exercise, is common among adolescents. The prevalence of EIB reportedly varies between 7–19 % in a general population of adolescents [1, 2] and to occur in a majority of patients with asthma [3]. Two population based studies reported the prevalence of EIB to be higher in girls than in boys [1, 4], whereas other studies did not observe any gender differences [5, 6].

We recently reported that adolescent females with EIB exhibited more exercise-induced dyspnoea than males [2] and that adolescents with exercise-induced dyspnoea suffer from more daytime and nocturnal dyspnoea, and absences from school compared with healthy controls [7]. Hallstrand et al. [8] observed that adolescents with dyspnoea secondary to exercise experienced lower health related quality of life (HRQoL), a finding evident both in subjects with and without asthma. Furthermore, children and adolescents with asthma have impaired HRQoL compared with control subjects [9]; among adolescents with asthma, the coexistence of EIB causes greater impairment of HRQoL [10]. To the best of our knowledge there are no previous studies investigating the association between EIB and health related quality of life (HRQoL) in a general population from a gender perspective.

Symptoms of EIB include cough, wheeze, chest tightness, and either shortness of breath or dyspnoea [3]. Billen et al. noted the possibility that subjects with EIB avoid physical activity and exercise due to fear of triggering symptoms [11]; one study noted lower levels of physical activity among children with EIB [12]. Physical activity levels are reportedly lower in females than in males in both general and asthmatic populations [13–16]. However, previous studies investigating gender differences in physical activity among adolescents with and without EIB are scarce.

The aim of this study was to study the relationship between EIB and HRQoL and EIB and physical activity levels in female and male adolescents with and without EIB.

## Methods

From a survey investigating the prevalence of self-reported exercise-induced dyspnoea subjects, 13–15 years, were recruited [7]. Classification was based on the question, “Have you had an attack of shortness of breath that came following strenuous activity at any time, in the last 12 months?”. A total of 199 adolescents with exercise-induced dyspnoea and 123 healthy controls were randomly selected and invited to participate in the study. The first 94 adolescents with exercise-induced dyspnoea and the first 46 healthy controls who agreed to participate were recruited. In total 140 adolescents participated in the study. The exclusion criteria included pulmonary diseases, apart from asthma, cardiac co-morbidity or an inability to perform exercise tests.

The subjects visited our clinic twice [2]. The first visit consisted of an interview regarding asthma, rhinitis and medications, and the adolescents completed questionnaires regarding health-related quality of life and anxiety and depression. The subjects’ heights and weights were measured, and venous blood samples for the analysis of IgE were collected. The second visit consisted of a standardised exercise challenge test on a treadmill with inhalation of dry air to detect EIB [2, 3]. Of the 140 subjects, 49 had EIB, and 91 did not have EIB. Following the EIB test, the participants were instructed to use an accelerometer to measure physical activity continuously for seven days.

### Exercise-induced bronchoconstriction test (EIB test)

The participants were instructed to withdraw short acting beta-2-agonists 8 h before the test, long acting beta-2-agonists 24 h before the test and leukotriene receptor antagonists 72 h before the test. They were also instructed not to use inhaled corticosteroids on the day of the test and to avoid vigorous exercise, heavy meals, nicotine and caffeine containing foods or beverages within four hours of the test.

Baseline spirometry was performed according to the guidelines from the European Respiratory Society (ERS) and the American Thoracic Society (ATS) [17], and the pre-challenge forced expiratory volume in one second (FEV_1_) was documented as the best FEV_1_ of three measurements (Cardio Perfect dynamic spirometry, Welch Allyn, Höganäs, Sweden). During the standardised six-minute treadmill exercise test (GE Marquette Series 2000 Treadmill, Waukesha WI, USA) each subject wore a nose clip and breathed through a tube (Aiolos Asthma test, Aiolos Medical AB, Karlstad, Sweden) connected to a central gas container with dry air (H_2_O/L < 5 mg/L, 18–22 °C). The subjects’ heart rates were continuously monitored using a heart monitor (The CASE Exercise Testing System, GE Medical Systems, Milwaukee, WI, USA). The objective was to increase cardiac frequency to 90 % of the predicted maximum ((208 − (0.7 × age)) × 0.9)) [18] within the first 1.5 min and maintain this level throughout the six minute test by adjusting the treadmill speed and slope. FEV_1_ was measured at 5, 10, 15 and 30 min following the test. The best FEV_1_ value of the two measurements at each time point was documented. EIB was defined as a decrease of ≥10 % in FEV_1_ from baseline [19].

### Health-related quality of life

The participants were asked to complete the teen version (ages 13 to 18 years) of the Pediatric Quality of Life Inventory Generic Core Scales instrument (PedsQL™ version 4.0) [20]. The PedsQL is a 23-items scale covering the following five health domains: physical function, emotional function, social function, school function and general well-being. The instrument yields three summary scores, as follows: a total scale score (23 items), a physical health summary score (8 items) and a psychosocial health summary score (15 items). A 5-point response scale was used (0 = never a problem; 1 = almost never a problem; 2 = sometimes a problem; 3 = often a problem; and 4 = almost always a problem). The items were reverse-scored and linearly transformed to a 0–100 scale (0 = 100, 1 = 75, 2 = 50, 3 = 25, 4 = 0); higher scores were indicative of better HRQoL. The Swedish version of PedsQL has acceptable reliability and validity [21].

### Hospital Anxiety and Depression Scale (HADS)

The Hospital Anxiety and Depression Scale (HADS) is a fourteen-item scale that generates ordinal data [22]. Seven of the items pertain to anxiety, and seven, to depression. Each item is rated on a 4-point scale as follows: 0 = not at all; 1 = sometimes; 2 = often; and 3 = all the time, for maximum subscale scores of 21 for anxiety and depression, respectively. HADS yields clinically meaningful results as a psychological screening tool in clinical group comparisons and correlation studies and addresses several aspects of disease and quality of life [23]. Regarding the validation of the questionnaire, a score >7 in the two subscales distinguishes non cases from suspected cases. The questionnaire has adequate test-retest reliability and distinguishes between adolescents diagnosed with either depression or anxiety and adolescents without these diagnoses [24].

### Symptoms and disorders

#### Exercise-induced dyspnoea

A positive answer to the following question: “Have you had an attack of shortness of breath that came following strenuous activity at any time, in the last 12 months?”

#### Ever asthma

A positive answer to the following question: “Have you ever been diagnosed as having asthma by a doctor?”

#### Rhinitis

A positive answer to the following question: “Have you ever had hay fever?”

#### Disturbed sleep

A positive answer to the following question: “Have you woken up several times at night, three or more nights per week during the last three months?”

### IgE sensitisation

IgE levels were measured against a mix of aeroallergens (animal, mite, mould, grass, tree and weed pollen (Phadiatop; ImmunoCAP; Thermo Fisher Scientific, Uppsala, Sweden) for all subjects. Subjects were diagnosed as atopic if they had IgE antibody levels ≥0.35 kU/L against the allergens in the panel.

#### Physical activity

The adolescents wore an accelerometer (Actical™ Mini Mitter CO, Bend, OR, USA) over their right ankle for seven consecutive days and nights with instructions to remove the device only when bathing or showering. The accelerometer (dimensions: 2.8 × 2.7 × 1.0 cm; weight: 17 g) measured and recorded time-stamped acceleration in all directions. The monitors collected data in 60-s intervals. The participants were blinded to the data while wearing the devices. Following the seven-day period, the monitors were collected, and the data were downloaded and archived.

The raw accelerometer data files were visually inspected for compliance and data integrity using the manufacturer's software (Actical V2.0, Mini Mitter Co., Inc.). Each accelerometer file had to satisfy the following criteria before it underwent further processing: the subject must have worn the monitor for at least four full days during the specified period; a “full day of wearing” was defined as at least 10 h of continuous monitoring from the first to the last bursts of activity data. Cut off points developed by Heil et al. [25] were used to calculate the time spent engaged in moderate to vigorous physical activity (MVPA). For each respondent, the minutes of MVPA were summed for each day and averaged for valid days. According to international guidelines regarding physical activity, children and adolescents should be engaged in MVPA for at least 60 min per day [26].

### Statistical analysis

The continuous variables were summarised either as the means and standard deviations (SD)/min and max (age, BMI, FVC, FEV_1_, PedsQL [27] and MVPA) or as medians and interquartile ranges (IQR) (HAD). The categorical variables (gender, overweight, exercise-induced dyspnoea, ever asthma, rhinitis, atopy, disturbed sleep and asthma medication) were summarised as numbers and percentages. Age, BMI, FVC and FEV_1_, HRQoL, anxiety and depression scores and physical activity levels were compared between the groups using either the unpaired Student t test or the Mann-Whitney U-test. For all categorical variables, a cross-tabulation vs. subject groups was performed, and the subject groups were compared using either the Pearson χ2 test or the Fisher’s exact test. A univariate linear regression analysis (with 95 % CI) was performed, with PedsQL (total and physical scores) and FEV_1_ serving as the dependent variables and EIB serving as the independent variable, both unadjusted and adjusted for the variable ever asthma and stratified for gender. Insignificant variables were removed from the model to obtain the simplest model with greatest explanatory power. Furthermore a multivariate regression model was performed, with PedsQL (Physical domain) as the dependent variable and gender, EIB, FEV_1_ %, asthma and anxiety as independent variables and the model were extended to include an interaction term between gender and EIB. The results were considered statistically significant at *p* < 0.05. All data manipulation and analysis were performed using Statistical Package for Social Science (SPSS) software, version 21 (SPSS Inc. Chicago, IL, USA).

## Results

A total of 49 (36 females) of the 140 adolescents had a fall ≥10 % in FEV_1_ after the exercise challenge test and were subsequently categorized as having EIB.

A larger proportion of females was observed in the group with EIB compared with the group without EIB (Table 1). Exercise-induced dyspnoea, asthma, sleep disturbances and asthma medication usage were more common among the subjects with EIB. The baseline FEV_1_ values were lower in the group with EIB compared with the group without EIB and the participants with EIB exhibited higher anxiety scores (Table 1).

**Table 1:** Characteristics of participants with and without a positive EIB test, *n* = 140. Data are presented as n (%) or mean ± SD unless otherwise indicated

	EIB *n* = 49	No EIB *n* = 91	*p*-value
Age (years), mean (min,max)	14.3 (13,15)	14.2 (13,15)	0.31
Female	36 (73)	48 (53)	0.017
BMI	21.3 ± 2.8	20.8 ± 2.9	0.30
Overweight and obesity	6 (12)	9 (10)	0.67
Exercise-induced dyspnoea	42 (86)	52 (57)	<0.001
Ever asthma^a^	22 (45)	18 (20)	0.002
Rhinitis	17 (35)	30 (33)	0.84
FVC% predicted^b^	93 ± 11	95 ± 11	0.31
FEV_1_ % predicted^b^	90 ± 9	94 ± 11	0.049
IgE sensitisation	21 (43)	41 (45)	0.80
Inhaled corticoid steroids	14 (28.6)	11 (12.1)	0.015
Short acting beta-2-agonist	22 (44.9)	22 (24.2)	0.012
Disturbed sleep	9 (18.4)	1 (1.1)	<0.001
HAD			
Anxiety, median (IQR)	6 (5)	4 (4)	0.007
Depression, median (IQR)	2 (4)	2 (3)	0.78

*EIB* exercise-induced bronchoconstriction, *BMI* body mass index (kg/m^2^), *HAD* Hospital anxiety and depression scale

^a^Self-reported, having ever been diagnosed by a physician as having asthma

^b^Forced vital capacity (FVC) and FEV_1_ presented as percentage of predicted recorded at baseline before EIB test

**Fig. 1:**
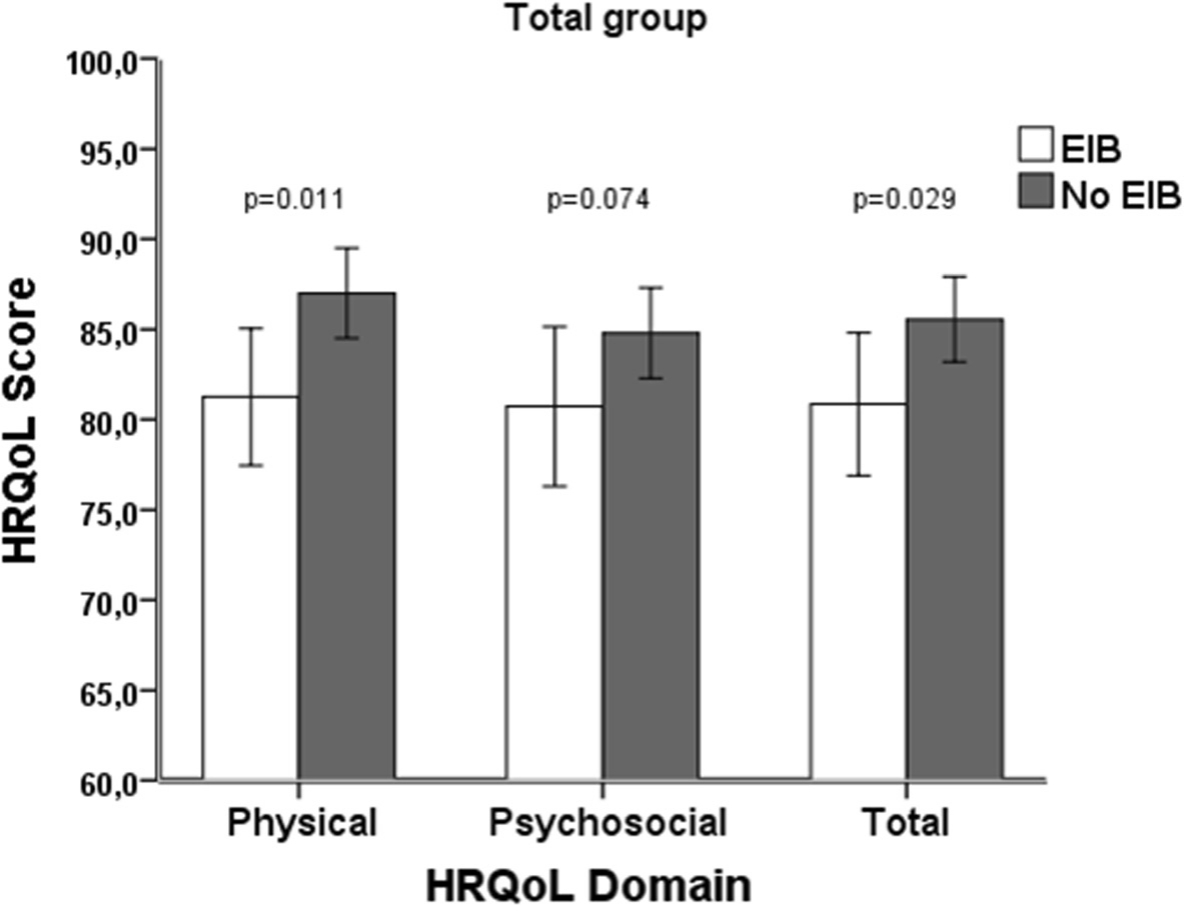
Comparison of HRQoL domain and total score in the total group of adolescents with and without EIB. Error bars represent standard error of mean

The group with EIB had lower HRQoL total scores and physical function scores compared with the participants without EIB (Fig. 1). There was no significant difference in the average minutes/day spent engaged in MVPA between the adolescents with and without EIB, nor was there any difference in the proportions of adolescents with and without EIB who adhered to the above recommendations regarding daily physical activity (27 vs. 39 %, *p* = 0.18).

### Stratification by gender

Among the females with EIB, a larger proportion of the group exhibited exercise-induced dyspnoea, asthma, and sleep disturbances and used asthma medications compared with the females without EIB (Table 2). The females with EIB also exhibited lower baseline FEV_1_ values compared with the females without EIB. The females with EIB exhibited a significantly higher anxiety score compared with the females without EIB; however, there was no difference between the groups with respect to the depression score.

**Table 2:** Characteristics of females with and without EIB and males with and without EIB. Data are presented as mean ± SD or n (%) unless otherwise indicated

	Female EIB *N* = 36	Female No EIB *N* = 48	*p-*value	Male EIB *N* = 13	Male No EIB *N* = 43	*p-*value
BMI	21.7 ± 3.0	20.5 ± 2.9	0.086	20.5 ± 1.7	21 ± 3.1	0.53
Overweight and obesity	6 (16.7)	4 (8.3)	0.31	0	5 (11.6)	0.58
Exercise-induced dyspnoea	33 (91.7)	26 (54.2)	<0.001	9 (69.2)	26 (60.5)	0.58
Ever asthma^a^	17 (47.2)	7 (14.6)	0.001	5 (38.5)	11 (28.9)	0.37
Rhinitis	11 (30.6)	15 (31.3)	0.95	6 (46.2)	15 (34.9)	0.46
FVC% predicted^b^	91.1 ± 10.6	94.0 ± 11.7	0.22	98.4 ± 10.5	96.2 ± 11.7	0.54
FEV_1_ % predicted^b^	88.8 ± 8.4	94.4 ± 10.6	0.012	94.5 ± 10.9	93.6 ± 10.5	0.74
IgE sensitisation	13 (36.1)	17 (35.4)	0.95	8 (61.5)	24 (55.8)	0.72
Inhaled corticoid steroids	11 (30.1)	5 (10.4)	0.020	3 (23.1)	6 (14.0)	0.42
Short acting beta-2-agonist	19 (52.8)	11 (22.9)	0.005	4 (30.8)	12 (27.9)	0.84
Disturbed sleep	8 (22.2)	0	0.001	1 (7.7)	1 (2.3)	0.41
HAD						
Anxiety, median (IQR)	7 (4)	4 (6)	0.031	3 (4)	3 (4)	0.72
Depression, median (IQR)	2 (4)	2 (3)	0.74	1 (4)	1 (4)	0.25

*EIB* exercise-induced bronchoconstriction, *BMI* body mass index (kg/m^2^), *HAD* Hospital anxiety and depression scale

^a^Self-reported, having ever been diagnosed by a physician as having asthma

^b^Forced vital capacity (FVC) and FEV_1_ presented as percentage of predicted recorded at baseline before EIB test

Among the males, there were no differences in any of the variables investigated between the subjects with and without EIB (Table 2).

**Fig. 2:**
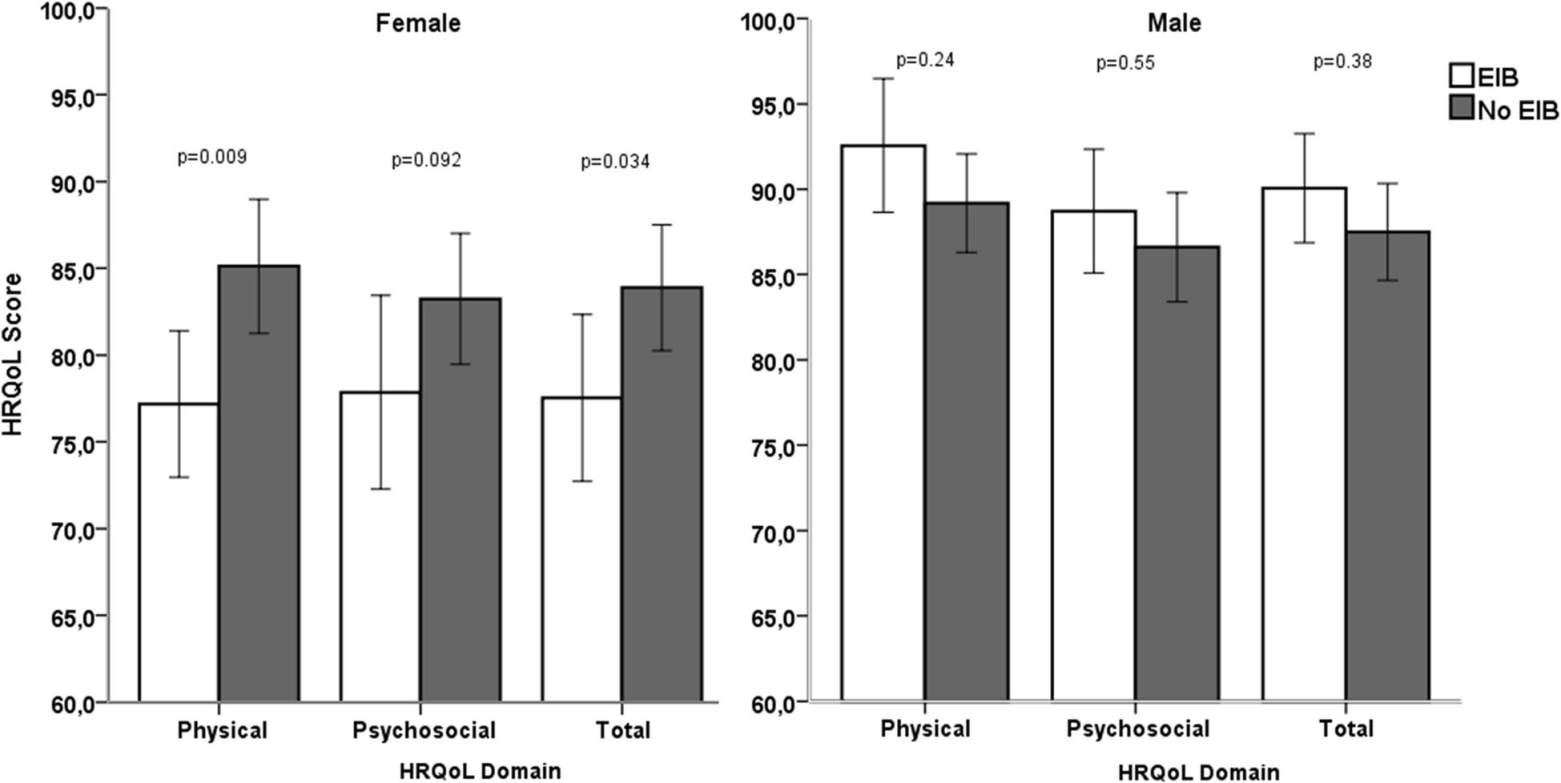
Comparison of HRQoL domain and total score in female and male adolescents with and without EIB. Error bars represent standard error of mean

The female group with EIB experienced significantly lower HRQoL, including lower total scores and physical function scores, compared with the females without EIB. By contrast, no differences were observed in HRQoL between the males with and without EIB (Fig. 2).

A subset of 130 of the participants (78 females and 52 males) exhibited accelerometer data that fulfilled the quality criteria and were subsequently analysed for minutes of MVPA.

**Fig. 3:**
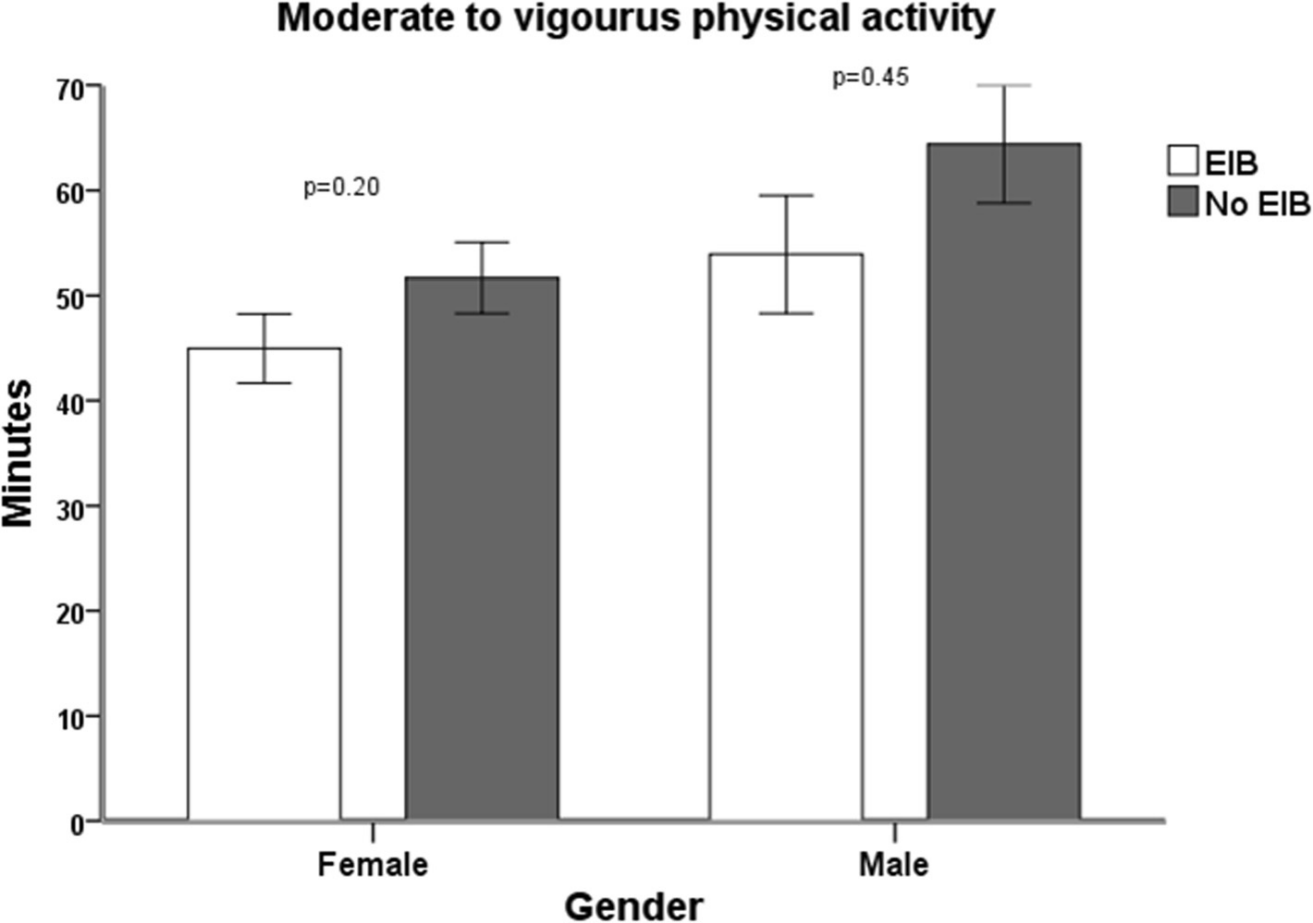
Comparison of average minutes of moderate to vigorous physical activity in female and male adolescents with and without EIB. Error bars represent standard error of mean

When comparing the females with and without EIB, there was no significant difference in the average min/day spent engaged in MVPA or the proportions of participants with MVPA values ≥60 min/day (20 vs. 33 %, *p* = 0.21). In males, there were no differences in average min/day spent engaged in MVPA, nor were there any differences in the proportions of participants meeting the recommendations regarding daily physical activity (46 vs. 46 %, *p* > 0.99) (Fig. 3).

### Univariate and multivariate linear regression analysis

Among the females enrolled in this study, EIB correlated significantly with both a lower total score and a lower physical function score in HRQoL in the univariate analysis. Following an adjustment for asthma, said correlation was no longer significant. However there were a statistically significant interaction with gender (*p* = 0.032) on the effect of EIB on the physical domain of HRQoL between adolescents with and without EIB even when performing adjustments for ever asthma and anxiety, e.g. females with EIB had lower HRQoL than counterparts without EIB while the opposite was seen for males.

There was a significant correlation noted between EIB and low FEV_1_ values in females in both the univariate and the multivariate analyses. Among the males, there was no correlation between the two variables (Table 3).

**Table 3:** Association between the dependent variables HRQoL and FEV_1_ % and the independent variable EIB, unadjusted and adjusted for the variable ever asthma, stratified for gender

	Female		Male	
	Independent variable EIB			
Dependent variable	Unadjusted	Adjusted for ever asthma	Unadjusted	Adjusted for ever asthma
	B (95 % CI)	B (95 % CI)	B (95 % CI)	B (95 % CI)
HRQoL				
Total score	−6.60 (−12.7 to −5.3)	−3.79 (−10.1 to 2.5)	2.50 (−3.2 to 8.1)	2.88 (−2.8 to 8.6)
Physical score	−8.07 (−14.0 to −2.1)	−5.6 (−11.8 to 0.62)	3.51 (−2.4 to 9.4)	3.74 (−2.2 to 9.7)
FEV_1_ %	−5.57 (−9.8 to −1.3)	−4.77 (−9.4 to −0.15)	1.10 (−5.6 to 7.8)	2.20 (−4.2 to 8.6)

*HRQoL* Health related quality of life, *FEV*_*1*_ *%* FEV_1_ presented as percentage of predicted recorded at baseline before EIB test, *EIB* Exercise-induced bronchoconstriction, *B* regression coefficient, *CI* confidence interval, *Ever asthma* Self-reported, having ever been diagnosed by a physician as having asthma

## Discussion and conclusion

The female adolescents with EIB exhibited lower HRQoL and lower lung function compared with the females without EIB; however, these differences were not observed in the males. EIB does not appear to affect physical activity levels in adolescents, as we observed no differences in the minutes of moderate to vigorous daily physical activity between the adolescents with and without EIB.

In our study, the adolescents with EIB exhibited lower total and physical function HRQoL scores compared with the subjects without EIB. Hallstrand et al. [8] studied adolescent athletes who experienced symptoms of dyspnoea during exercise and observed that they exhibited significantly lower total and physical function HRQoL scores compared with adolescents without dyspnoea. However, after verifying the diagnoses of EIB with a free-running test, the authors did not observe a difference in HRQoL between the adolescents with and without EIB. One possible explanation for the contrasting results between our study and the study by Hallstrand is that they did not use a standardised exercise test controlling for pulse and the relative humidity of the inspired air, which may have resulted in a number of false negative EIB tests among the adolescents with exercise-induced dyspnoea, which affected the subsequent analysis.

The present study was the first to investigate gender differences regarding the relationship between EIB and HRQoL. Our findings indicated that female adolescents with EIB exhibited significantly lower total and physical HRQoL compared with females without EIB, differences that were not observed in males. Consistent with our results, Varni et al. [28] observed similar results in HRQoL among subjects with mild persistent asthma when using the PedsQL to investigate the effects of paediatric chronic diseases. In our study, the relationship between EIB and the low HRQoL observed in females was partially explained by the higher prevalence of asthma among the female subjects with EIB compared with the male subjects. After adjusting for asthma, the results indicate that EIB is more strongly associated with asthma. However, there was a significant gender interaction in the comparison of the physical domain of HRQoL between adolescents with and without EIB even when performing adjustments for asthma. Female sex is reportedly an independent determinant of a lower quality of life in subjects with asthma [29]. Therefore, interventions targeting asthma in females with EIB may have an effect on HRQoL in this group.

The females with EIB exhibited lower baseline lung function values and reported more sleep disturbances compared with the females without EIB. By contrast, there were no differences between the males with and without EIB. Among the females enrolled in this study, there was a significant relationship between EIB and lower baseline FEV_1_ values, a relationship that persisted following adjustment for asthma. This relationship was not observed among the male subjects. A relationship between lower baseline lung function and asthma-like symptoms has been described in elite female ice-hockey players [30]. However, following standardised exercise testing for EIB, the authors observed no relationship between baseline lung function and EIB. We believe that our results regarding the relationship between lung function and EIB are novel but should be investigated further, as previous studies did not report the same results [30, 31].

We observed no difference in psychosocial HRQoL between the adolescents with and without EIB among either the female subjects or the male subjects. However, the females with EIB exhibited significantly higher anxiety scores compared with the females without EIB. Adolescent asthmatics are reportedly at higher risk of developing anxiety compared with healthy control subjects [32], and adolescents suffering from asthma and anxiety disorders are significantly more likely to be female [33].

The present study observed no difference in daily physical activity levels or the proportion of adolescents meeting the recommendations regarding daily physical activity between the adolescents with and without EIB. Nor were there any differences noted in physical activity level between the females and males with and without EIB. These results were consistent with the observations of previous studies in which children with newly diagnosed asthma and children exhibiting symptoms of asthma were reportedly as physically active as their healthy peers [34, 35]. Some studies investigating children and adolescents with asthma have not demonstrated differences in physical activity levels between said children and adolescents and their non-asthmatic peers [36, 37], whereas others have suggested that children and adolescents with asthma are less physically active [38, 39]. It has been argued that physical activity rates in children and adolescents with asthma may appear artificially high because active children and adolescents are more likely to be diagnosed with asthma and EIB [40]. It has been reported that children and adolescents with asthma believe that their levels of physical activity are reduced as a result of their disease [41]; a large population based Greek study [12] observed that 10–12-year-old children who tested positive for EIB reported spending shorter amounts of time engaged in daily moderate physical activity compared with control subjects, which raises the question of how to measure physical activity levels; one could speculate that among subjects with EIB that self-reported levels of physical activity may differ from objectively measured levels.

A strength of the present study was the standardised exercise test with dry air inhalation to verify the diagnoses of EIB, as well as the use of accelerometers to objectively measure physical activity levels. The use of an accelerometer does not allow for the recording of data pertaining to swimming or other water sports or activities, however. Additionally, the cross-sectional design of our study did not establish clear casual relationships.

In conclusion, the female adolescents with EIB experienced lower physical HRQoL and exhibited poorer lung function compared with the females without EIB, differences that were not observed among the males with and without EIB. The reason for this discrepancy may be that EIB is more strongly associated with asthma among females than among males. The female subjects with EIB also experienced greater anxiety and exhibited lower baseline lung function and more frequent sleep disturbances compared with the females without EIB. EIB does not appear to affect daily physical activity levels, either in females or in males. Interventions targeting asthma in females with EIB may have an effect on the physical domain of HRQoL in this group.

### Ethics approval and consent to participate

Ethical approval was obtained from the Uppsala Local Ethics Committee, Sweden (Reference 2011/413). All participants, as well as their guardians, provided written informed consent.

### Consent for publication

Not applicable.

### Availability of data and materials

The datasets supporting the conclusions of this article are available upon request to the corresponding author.

## Abbreviations

ATS: American Thoracic Society
BMI: body mass index
EIB: exercise-induced bronchoconstriction
ERS: European Respiratory Society
FEV_1_: forced expiratory volume in one second
FVC: forced vital capacity
HADS: Hospital anxiety and depression scale
HRQoL: health related quality of life
MVPA: moderate to vigorous physical activity
PedsQL: Pediatric Quality of Life Inventory
